# Relationship between binocular vision and Govetto’s stage in monocular idiopathic epiretinal membrane

**DOI:** 10.1038/s41598-024-71594-x

**Published:** 2024-09-03

**Authors:** Kanae Tsuda, Manabu Miyata, Kentaro Kawai, Shinya Nakao, Akinari Yamamoto, Kenji Suda, Eri Nakano, Miho Tagawa, Yuki Muraoka, Ryo Sakata, Akitaka Tsujikawa

**Affiliations:** Department of Ophthalmology and Visual Sciences, Kyoto University Graduate School of Medicine, 54 Shogoin Kawahara-cho, Sakyo-ku, Kyoto City, Kyoto Prefecture 606-8507 Japan

**Keywords:** Binocular vision, Epiretinal membrane, Govetto’s stage, Foveal avascular zone, Diseases, Medical research

## Abstract

Govetto’s staging system (stages 1–4) for epiretinal membrane (ERM) based on optical coherence tomography images is a useful predictor of monocular visual function; however, an association between Govetto’s stage and binocular vision has not been reported. This study aimed to investigate the factors associated with Govetto’s stage among the monocular and binocular parameters. This retrospective study included consecutive patients with treatment-naïve eyes with unilateral ERM without pseudo-hole. We investigated Govetto’s stage, degrees of aniseikonia and metamorphopsia, foveal avascular zone area, central retinal and choroidal thickness, vertical ocular deviation, stereopsis, and binocular single vision (BSV). We compared the parameters between the BSV-present and BSV-absent groups and investigated correlations between Govetto’s stage and the monocular and binocular parameters. Twenty-eight eyes of 28 patients were examined (age, 66.6 ± 10.2 years). In multivariate correlation analyses, Govetto’s stage correlated with BSV (*P* = 0.04, β = − 0.36) and central retinal thickness (*P* < 0.001, β = 0.74). Of the eyes, 18 were assigned to the BSV-present group and 10 to the BSV-absent group. Govetto’s stage was significantly more advanced in the BSV-absent group than in the BSV-present group (3.2 ± 0.8 vs 2.5 ± 0.7, *P* = 0.03). Of the 28 patients, 11 (39%) showed small-angle vertical deviations (1–12Δ). In conclusion, our findings showed that Govetto’s stage correlated with binocular vision in patients with monocular ERM. Govetto’s staging is a useful parameter for predicting not only monocular but also binocular vision.

## Introduction

Epiretinal membrane (ERM) can cause monocular visual dysfunction, including a decline in best-corrected visual acuity (BCVA), metamorphopsia, and macropsia. A previous study of the central bouquet sign suggested that foveal Müller cells play an integral role in transmitting mechanical forces to the central foveal cones^1^, and the abnormality by ERM traction may subsequently induce these visual disturbances. A prevalence of 8.9–28.9% has been reported in large cohort and multi-ethnic studies^2–4^; and the risk factors for ERM were older age, diabetes mellitus, and cataract surgery. Aniseikonia caused by unilateral macropsia may result in decreased binocularity, including stereopsis^5^; however, it has been demonstrated that vitrectomy does not change the amount of aniseikonia^6^, in contrast to metamorphopsia^7^.

The diagnosis of ERM relies on clinical examination in combination with optical coherence tomography (OCT)^8^. Recently, Govetto et al. proposed an ERM staging system (stages 1–4) based on OCT images and showed that BCVA progressively declined from stage 1 to stage 4^9^. Several other studies have used Govetto’s staging system to analyze monocular visual function^10,11^. However, no study has investigated the correlation between Govetto’s stage and binocular vision.

Dragged-fovea diplopia which consists of central diplopia in the presence of peripheral fusion (central-peripheral rivalry-type diplopia) secondary to dragging of the fovea by ERM, has been described previously^12,13^. Therefore, prism therapy cannot satisfy patients with dragged-fovea diplopia. The dragged-fovea induced by ERM can lead to refractory small-angle vertical pseudo-strabismus. Particularly, an eye with a high Govetto’s stage of 3 or 4 has an ectopic inner foveal layer, which would induce dragged-fovea. Thus, Govetto’s stage might correlate with binocular function.

Here, we investigated the factors associated with Govetto’s stage among the monocular and binocular parameters because Govetto’s stage may reflect aniseikonia and dragged-fovea diplopia in monocular ERM, which can induce binocular visual dysfunction.

## Methods

This retrospective study was approved by the Ethics Committee of Kyoto University Graduate School of Medicine (Kyoto, Japan). All the study protocols adhered to the tenets of the Declaration of Helsinki, and all participants provided written informed consent for participation in this study.

### Participants

**Fig. 1:**
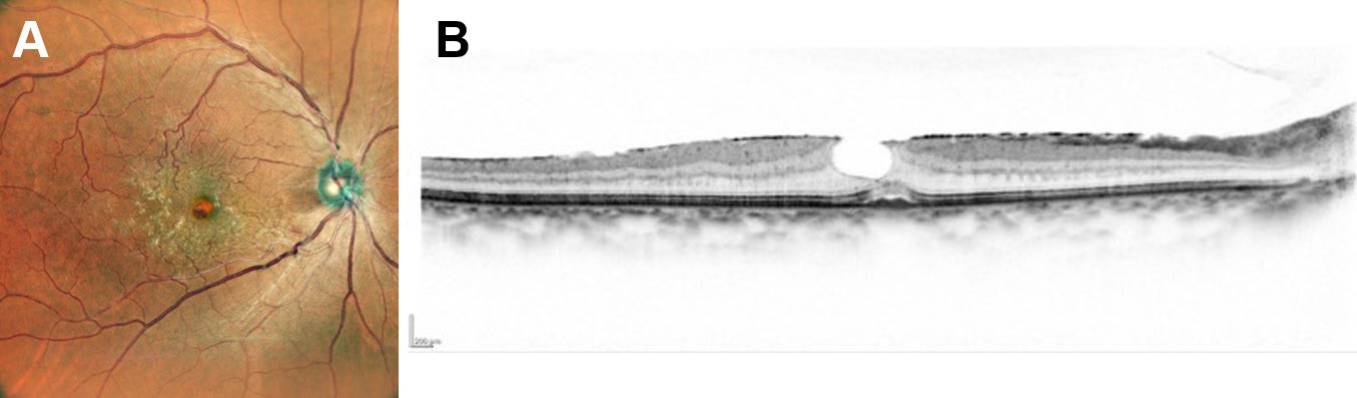
Representative images of scanning laser ophthalmoscope (SLO) fundus photography and optical coherence tomography (OCT) of an eye with a pseudo-hole. Images of the right eye of a man in his 60 s. Best-corrected visual acuity was 20/40. Aniseikonia was 4%. (**A**) SLO image. Retinal folds induced by epiretinal membrane (ERM) and a red spot at the fovea are observed. (**B**) OCT image. Absence of ERM only in the fovea and slight amount of subretinal fluid are observed. On applying Govetto’s staging, this eye is Stage 1. However, the visual function is relatively poor. Therefore, we excluded eyes with pseudo-holes from this study.

This study included consecutive patients with treatment-naïve eyes and with a diagnosis of unilateral ERM who visited Kyoto University Hospital between May 2018 and November 2021 and underwent aniseikonia and ocular positioning tests (detailed examinations are described hereinafter). The exclusion criteria were as follows: (1) either eye with vision-threatening macular disease, including retinal vein occlusion, proliferative diabetic retinopathy, retinitis pigmentosa, central serous chorioretinopathy, age-related macular degeneration, macular hole, glaucoma, or uveitis; (2) congenital strabismus with ≥ 20Δ; (3) pseudo-hole (Fig. 1); (4) BCVA < 20/20 in the healthy eye; and (5) inability to undergo the stereo test or ocular position test. These were determined by TK based on the medical records.

### Ophthalmological examination

All eyes underwent a comprehensive ophthalmological examination, including BCVA measurement with a Landolt chart, intraocular pressure measurement, slit-lamp biomicroscopy, indirect ophthalmoscopy, color fundus photography, and OCT. Retinal specialists diagnosed ERM based on the above multimodal imaging mainly by OCT, considering the presence of an irregular and hyper-reflective layer over the inner limiting membrane (ILM), often associated with signs of wrinkling of the underlying retina, and the frequent presence of hyporeflective spaces between the ERM and ILM^14^. Furthermore, axial length was measured by partial coherence interferometry and swept-source OCT angiography (PLEX Elite; Carl Zeiss Meditec, Dublin, CA, USA). Aniseikonia was assessed using the New Aniseikonia Test (Handaya Co., Tokyo, Japan) by the difference (%) in half-circle size seen by the affected and healthy eyes through red-green glasses. Metamorphopsia of the affected eye was assessed using M-Charts (Inami Co., Tokyo, Japan), consisting of 19 dotted vertical or horizontal lines with dot intervals ranging from 0.2 to 2.0 degrees in visual angle. Stereopsis and binocular single vision (BSV) were assessed using the TNO stereo test (Haag-Streit, Harlow, UK). The primary ocular position at far distance was determined using prisms and the alternate cover test while keeping the patient’s head perpendicular.

### Measurement of central retinal and choroidal thickness

One investigator (KT) measured the central retinal thickness (CRT; distance between the vitreoretinal surface and the outer border of the sensory retina) and central choroidal thickness (CCT; distance between the outer surface of Bruch’s membrane and the chorioscleral interface) at the fovea on horizontal and vertical B-scan OCT images through the fovea, using built-in OCT software, and calculated the average horizontal and vertical thicknesses for further analysis.

### Foveal avascular zone measurement

**Fig. 2:**
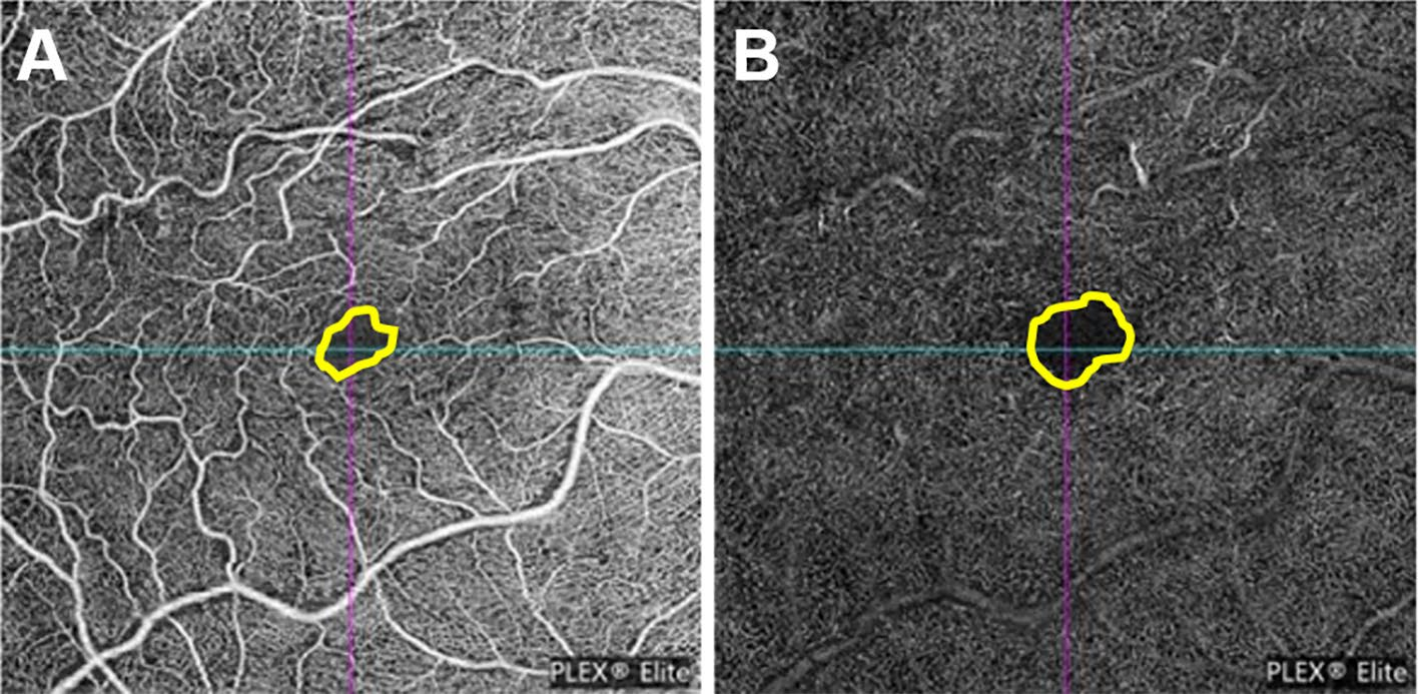
Foveal avascular zone (FAZ) measurement using optical coherence tomography angiography (OCTA) images. We manually delineated the boundary of the FAZ and measured the area of the FAZ for both eyes in en face images of the superficial (**A**) and deep plexus capillaries (**B**) using ImageJ software.

All 3 × 3 mm OCT angiography en face images centered on the fovea were acquired using the macular angiography protocol of the PLEX-Elite 9000 containing 500 × 500 A-scans. En face images of the superficial capillary plexus were segmented with the inner boundary at the ILM and the outer boundary at the inner plexiform layer (IPL). En face images of the deep capillary plexus were segmented with the inner boundary at the IPL and the outer boundary at the outer plexiform layer (OPL). We manually delineated the boundary of the foveal avascular zone (FAZ) and measured the area of the FAZ for both eyes in en face images of the superficial and deep plexus capillaries using ImageJ software (National Institutes of Health, Bethesda, Maryland, USA) (Fig. 2). Furthermore, we calculated the ratio of the FAZ area of the affected eye to the FAZ area of the healthy eye in each participant.

### Govetto’s staging system

Two trained ophthalmologists (KT and RS) independently staged ERM using OCT B-scan images through the fovea based on Govetto’s staging system, which contains stages 1, 2, 3, and 4^9^. Each stage is described as follows: stage 1 showed a foveal pit and well-defined retinal layers; stage 2 showed the absence of a foveal pit and well-defined retinal layers; stage 3 showed the absence of a foveal pit, well-defined retinal layers, and an ectopic inner foveal layer (the presence of a continuous hyporeflective or hyperreflective band, extending from the inner nuclear layer and IPL across the foveal region); and stage 4 showed the absence of a foveal pit, disrupted retinal layers, and ectopic inner foveal layers. In cases with grading discrepancies, a third retinal specialist (MM) made the final judgment.

### Group assignment

Based on the results of Plate IV of the TNO stereo test, the participants were divided into two groups: BSV-present and BSV-absent. Plate IV involves three circles of red, grey, and green seen through red-green glasses; therefore, it can assess binocularity, including diplopia, suppression, or BSV. Specifically, patients in the BSV-present group responded by seeing all three dots, whereas those in the BSV-absent group responded by seeing two dots (suppression) or four dots (diplopia). Furthermore, patients who correctly identified Plates I, II, and III in the TNO test were considered to have stereopsis.

### Statistical analysis

Data are presented as mean ± standard deviation, where applicable. We converted BCVA into the logarithm of the minimum angle of resolution (logMAR) values for statistical analysis. The average of the vertical and horizontal M-Chart scores was used as the representative metamorphopsia score, in accordance with a previous report^9^. Comparative analyses between the BSV-present and BSV-absent groups were performed using the Mann–Whitney U test or chi-square test where applicable. Univariable correlation analyses of Govetto’s stage with the studied parameters were performed using Spearman’s rank correlation coefficient. Multivariable correlation analyses were performed using Govetto’s stage as the dependent variable and the studied parameters with *P*-values of < 0.10 in Spearman’s correlation test as the independent variables. *P*-values of < 0.05 were considered statistically significant. When data were missing, we excluded them from each analysis. All statistical analyses were performed using SPSS version 27 software (IBM Corp., Armonk, NY, USA).

## Results

We evaluated consecutive 28 eyes of 28 patients who met the criteria (age, 66.6 ± 10.2 years; 16 men; logMAR BCVA in the affected eyes, 0.10 ± 0.13) (Table 1). Aniseikonia was 5.0 ± 3.4%, and all patients showed macropsia in the affected eye. Univariable correlation analyses revealed that Govetto’s stage correlated with logMAR BCVA (*P* = 0.02, r = 0.45), stereopsis (*P* = 0.046, r = − 0.38), BSV (*P* = 0.03, r = − 0.42), and CRT (*P* = 0.001, r = 0.60) (Table 2). In the multivariate correlation analyses, Govetto’s stage correlated with BSV (*P* = 0.04, β = − 0.36) and CRT (*P* < 0.001, β = 0.74). Furthermore, aniseikonia did not correlate with BSV (*P* = 0.30) or stereopsis (*P* = 0.86); M-chart scores also did not correlate with BSV or stereopsis (*P* = 0.40 and *P* > 0.999, respectively).

**Table 1:** Comparison of baseline parameters between the BSV-present and BSV-absent groups.

	All patients (n = 28)	BSV-present group (n = 18)	BSV-absent group (n = 10)	*P*-value
Age, years	66.6 ± 10.2	64.1 ± 10.6	71.2 ± 8.2	0.03*
Sex (male/female)	16/12	10/8	6/4	0.82
Aniseikonia, %	5.0 ± 3.4	5.6 ± 3.5	3.9 ± 3.0	0.29
Average M-chart score, degrees	0.59 ± 0.51	0.52 ± 0.36	0.71 ± 0.73	1.00
LogMAR BCVA	0.10 ± 0.13	0.09 ± 0.12	0.12 ± 0.15	0.87
Axial length^a^, mm	24.64 ± 1.97	25.04 ± 1.87	23.82 ± 2.03	0.14
Horizontal deviation, degrees (exo-, − ; eso-, +)	− 1.77 ± 2.40	− 2.25 ± 2.40	− 0.92 ± 2.26	0.10
Vertical deviation, degrees	0.71 ± 1.49	0.79 ± 1.70	0.57 ± 1.08	0.98
Stereopsis (present/absent)	15/13	15/3	0/10	< 0.001*
CRT^b^, μm	447.4 ± 155.5	446.3 ± 167.5	449.4 ± 141.4	0.58
CCT^c^, μm	331.9 ± 93.6	338.6 ± 92.7	320.6 ± 99.0	0.58
Superficial FAZ area in the affected eye^d^, mm^2^	0.050 ± 0.069	0.061 ± 0.078	0.025 ± 0.036	0.20
Deep FAZ area in the affected eye^e^, mm^2^	0.17 ± 0.23	0.20 ± 0.27	0.10 ± 0.07	0.81
Superficial FAZ ratio (affected/healthy)^f^, %	25.06 ± 36.52	31.82 ± 43.11	12.53 ± 15.02	0.36
Deep FAZ ratio (affected/healthy)^g^, %	23.04 ± 18.06	25.00 ± 20.84	19.38 ± 11.84	0.78
Govetto stage (1–4)	2.8 ± 0.8	2.5 ± 0.7	3.2 ± 0.8	0.03*

Data are presented as means ± standard deviations.

BSV-present group = Patients in whom binocular single vision was assessed using a TNO stereo test.

BSV-absent group = Patients in whom binocular single vision was not assessed using a TNO stereo test.

logMAR BCVA = logarithm of the minimal angle of resolution best-corrected visual acuity; CRT = central retinal thickness; CCT = central choroidal thickness; FAZ = foveal avascular zone.

*Statistically significant (*P* < 0.05).

The data for ^a^, ^b^, ^c^, ^d^, ^e^, ^f^, and ^g^ are missing in 1, 1, 1, 6, 6, 8, and 8 eyes, respectively.

**Table 2:** Correlation of the studied parameters with the Govetto stage.

	Univariable Analysis		Multivariable Analysis	
	*P*	r	*P*	β
Age	0.67	0.08	–	–
Sex (1, male; 2, female)	0.81	− 0.05	–	–
Aniseikonia	0.43	− 0.16	–	–
Average M-chart score	0.11	0.31	–	–
LogMAR BCVA	0.02*	0.45	0.31	0.16
Axial length^a^	0.10	− 0.32	–	–
Horizontal deviation (exo-, − ; eso-, +)	0.26	0.22	–	–
Vertical deviation	0.76	− 0.06	–	–
Stereopsis (0, absent, 1, present)	0.046*	− 0.38	N/A	N/A
Binocular single vision (0, absent; 1, present)	0.03*	− 0.42	0.04*	− 0.36
CRT^b^	0.001*	0.60	< 0.001*	0.74
CCT^c^	0.92	0.02	–	–
Superficial FAZ area in the affected eye^d^	0.19	− 0.29	–	–
Deep FAZ area in the affected eye^e^	0.26	− 0.25	–	–
Superficial FAZ ratio (affected/healthy)^f^	0.09	− 0.39	0.18	− 0.21
Deep FAZ ratio (affected/healthy)^g^	0.20	− 0.30	–	–

logMAR BCVA = logarithm of the minimal angle of resolution best-corrected visual acuity; CRT = central retinal thickness; CCT = central choroidal thickness; FAZ = foveal avascular zone; N/A = not available.

The data for ^a^, ^b^, ^c^, ^d^, ^e^, ^f^, and ^g^ are missing in 1, 1, 1, 6, 6, 8, and 8 eyes, respectively.

*Statistically significant (*P* < 0.05).

Of the patients, 18 (64%) and 10 (36%) were assigned to the BSV-present and BSV-absent groups, respectively. The age of the patients was significantly lower in the BSV-present group than that in the BSV-absent group (64.1 ± 10.6 vs 71.2 ± 8.2 years, *P* = 0.03). Additionally, 15 of the 18 patients (83%) in the BSV-present group had stereopsis, whereas none of the 10 patients in the BSV-absent group had stereopsis. The stereopsis rate was significantly higher in the BSV-present group (*P* < 0.001). Govetto’s stage was significantly advanced in the BSV-absent group, compared with that in the BSV-present group (3.2 ± 0.8 vs 2.5 ± 0.7, *P* = 0.03). The other parameters, including sex, aniseikonia, M-chart score, logMAR BCVA, axial length, horizontal deviation, vertical deviation, CRT, CCT, or FAZ area did not significantly differ between the groups. Furthermore, 11 of the 28 patients (39%) showed small-angle vertical deviations (1–12Δ), including seven (39%) and four patients (40%) in the BSV-present and absent groups, respectively. This may be attributed to dragged-fovea pseudo-strabismus resulting from ERM-induced traction on the macula. However, 6 of 10 patients in the BSV-absent group showed no ocular deviation. The data for axial length, CRT, CCT, FAZ area, and FAZ area ratio were missing in 1, 1, 1, 6, and 8 eyes, respectively.

## Discussion

To the best of our knowledge, this is the first study to demonstrate a correlation of Govetto’s stage with both monocular and binocular visual disturbances in eyes with unilateral ERM. We found that patient age and Govetto’s stage were significantly higher and that rate of eyes with stereopsis was lower in the BSV-absent group, compared with those in the BSV-present group; however, aniseikonia and metamorphopsia were not significantly different between the two groups. Our findings suggest that Govetto’s stage is an important parameter that reflects both monocular and binocular visual disturbances.

Small-angle vertical deviation (≥ 1Δ) was observed in 39% of the eyes in the present study, indicating that vertical pseudo-strabismus (vertical transposition of the fovea dragged by ERM) is often present in unilateral ERM. Although this study did not perform the “lights on–off test”, which Guyton et al. reported as useful for diagnosing dragged-fovea diplopia syndrome^12,15^, this rate is much higher than the incidence rate of small-angle hypertropia reported in a population-based cohort study (annual, 7.5/100,000 people), even after considering the differences in the definitions^16^. We consider that the present study included many patients with pseudo-strabismus. However, no differences were observed in vertical deviations between the BSV-present and BSV-absent groups. Multiple other parameters might affect BSV in patients with monocular ERM.

A previous study showed that Titmus and TNO stereo test scores were significantly associated with the degree of aniseikonia^5^. However, our results suggested that aniseikonia was not significantly associated with BSV or stereopsis. Aniseikonia is difficult to estimate accurately. Another previous study suggested that the repeatability of the New Aniseikonia Test was not high (95% limits of agreement, ± 2%) and recommended caution when interpreting the results of this test^17^. Our results for aniseikonia using the New Aniseikonia Test might be inaccurate, particularly for patients with metamorphopsia and old age in the present study; therefore, we might find no correlation between aniseikonia and binocularity, which is unexpected.

This study has some limitations. First, the sample size was relatively small. We set strict exclusion criteria to accurately assess the correlation between binocular vision and monocular ERM-specific parameters using Govetto’s staging system. Second, aniseikonia was difficult to accurately assess in patients with monocular ERM. The New Aniseikonia Test used in the present study is based on adjusting the size of both the right and left half-circles. However, metamorphopsia in eyes with ERM may induce a change in the shape of the circle. The lack of correlation between aniseikonia and binocularity in the present study might have been caused by an inaccurate assessment of aniseikonia. Third, we used the TNO stereo test. A previous study showed that stereoacuity correlated with disease duration using the Titmus stereo test (Stereo Optical Co., Inc., Chicago, IL) in eyes with unilateral ERM (*P* = 0.045); however, no correlation was identified using the TNO stereo test (*P* = 0.21)^18^. Further research using the Titmus stereo test is also needed. Fourth, we assigned patients into the BSV-present or BSV-absent groups based on the results of TNO plate IV. This method cannot assess retinal rivalry or peripheral diplopia. The best method remains unclear.

In conclusion, our findings show that Govetto’s stage correlated with binocular vision in patients with monocular ERM. Govetto’s stage is a useful parameter for predicting not only monocular but also binocular vision.

## Data Availability

The datasets generated during and/or analyzed during the current study are available from the corresponding author on reasonable request.
